# Do ward changes affect outcomes differently in people living with dementia?

**DOI:** 10.1093/ageing/afaf372

**Published:** 2026-01-15

**Authors:** Emma Elliott, Robyn Hamilton, Luke Munford, Connor Richardson, Lindsey Darley, Rebecca Thompson, Daniel Rowbotham, Emma R L C Vardy

**Affiliations:** National Institute for Health and Care Research (NIHR) Applied Research Collaboration-Greater Manchester, Faculty of Biology, Medicine and Health, School of Health Sciences, The University of Manchester, Manchester M13 9PL, UK; Manchester Academic Health Science Centre, Manchester, M13 9NQ, UK; Northern Care Alliance NHS Foundation Trust, Salford, UK; National Institute for Health and Care Research (NIHR) Applied Research Collaboration-Greater Manchester, Faculty of Biology, Medicine and Health, School of Health Sciences, The University of Manchester, Manchester M13 9PL, UK; Manchester Academic Health Science Centre, Manchester, M13 9NQ, UK; National Institute for Health and Care Research (NIHR) Applied Research Collaboration-North East and North Cumbria, Newcastle University, Newcastle upon Tyne, NE4 5PL, UK; Northern Care Alliance NHS Foundation Trust, Salford, UK; Northern Care Alliance NHS Foundation Trust, Salford, UK; Northern Care Alliance NHS Foundation Trust, Salford, UK; National Institute for Health and Care Research (NIHR) Applied Research Collaboration-Greater Manchester, Faculty of Biology, Medicine and Health, School of Health Sciences, The University of Manchester, Manchester M13 9PL, UK; Manchester Academic Health Science Centre, Manchester, M13 9NQ, UK; Northern Care Alliance NHS Foundation Trust, Salford, UK

**Keywords:** dementia, ward changes, hospitalisation, ward moves, transfers, older people

## Abstract

**Background:**

Ward changes during hospital admissions are associated with poorer outcomes, but their impact on people living with dementia is unknown.

**Objective:**

To examine whether individuals with dementia are more adversely affected by ward changes.

**Setting:**

Four hospitals within a UK NHS Trust.

**Subjects:**

Individuals aged ≥65 years.

**Methods:**

This service evaluation included data from 01/2023–02/2024. Generalised estimating equations were used to fit linear and logistic regression models. Associations between ward changes, dementia status and their interaction on three outcomes—length of stay (LOS), inpatient mortality and discharged to a care home—were examined. Fully adjusted models accounted for demographic, socioeconomic factors and hospital site.

**Results:**

27,140 admissions, 19,392 unique individuals (2760 with dementia) were included. Mean age at first admission 79.0 (SD 8.1). In the fully adjusted LOS model, both ward changes (β = 5.2, *P* < .001) and the interaction with dementia (β = 1.7, *P* < .001) were associated with longer LOS. In the fully adjusted mortality model, dementia was associated with increased risk of mortality (OR = 1.4, *P* = .013) but there was no interaction effect of ward changes and dementia. In the fully adjusted care home admission model, dementia (OR = 4.4, *P* < .001) and ward changes (OR = 1.3, *P* < .001) were associated with increased risk, without evidence of interaction.

**Conclusions:**

Our results suggest that ward changes disproportionately affect LOS, but not mortality or discharge destination, in people living with dementia. Minimising ward transfers may improve outcomes for all older adults but is particularly important in dementia care to reduce the risk of extended LOS and potential associated inpatient harm.

## Key Points

- In hospitalized older adults, each ward change increased length of stay by 5 days and a further 1.7 days for those with dementia.
- There was no interaction between ward changes and dementia on mortality and new care home admission.
- Minimising ward changes should be prioritised in older adults and particularly in people living with dementia.

## Introduction

Ward changes or moves are not uncommon during hospital admissions, with some of the reasons for changes driven by bed availability, rather than clinical need. This can result in a lack of continuity of care and poorer outcomes for patients [1, 2]. Previous studies have found that room or ward changes in older people increases their length of stay [1], risk of delirium [3, 4] and risk of falls [5–7], but the interaction of the number of ward changes and having dementia has not been explored.

It is well known that hospitalisation is associated with challenges for people living with dementia, including longer hospital admissions [8], and higher rates of readmission, being discharged to a care home and mortality [9–11] compared to those without dementia, but we don’t know if ward changes are associated with these outcomes. There is rationale to hypothesise that people living with dementia would be more negatively affected by ward changes, e.g. they may experience increased confusion and disorientation [12], particularly because a change in environment can be a trigger for delirium [13]. This is why delirium prevention protocols recommend minimising room transfers [14, 15]. Improving dementia care in hospital is a key area for research and policy [16]. Whilst we would expect that moving people across the hospital multiple times could result in adverse outcomes, the full impact on this group is not yet known. Therefore, further evidence is needed to inform and promote changes in practice.

Some hospitals have implemented continuous flow models, whereby a set number of patients are moved at set times from the emergency department (ED) to inpatient wards [17]. This is to avoid patients having a prolonged stay in the ED but could result in patients being placed on a clinically inappropriate ward and may result in someone being placed on multiple wards during one hospital admission. There is evidence that people living with dementia and/or delirium admitted to wards that do not specialise in their primary health issue miss out on expertise input and have poorer outcomes [18].

This study aims to examine associations of the number of ward changes on length of stay, mortality and discharge destination and interactions of ward changes and dementia to examine whether individuals with dementia are more adversely affected by ward changes. We hypothesise that there would be an interaction effect between ward changes and dementia on at least one of the selected outcomes.

## Methods

This was an observational study using routinely collected, longitudinal data from four hospitals within a UK National Health Service (NHS) trust in Greater Manchester, England. The NHS Trust covers four localities (Salford, Bury, Oldham, Rochdale), serving a population of ~1 million people and covering areas affected by high levels of deprivation, according to the 2019 English Index of Multiple Deprivation (IMD) data [19] (the official measure of relative deprivation for small areas in England [See Appendix 1  for  Supplementary  methods]).

The NHS trust manages 2000 beds across four hospitals; three of the hospitals have type 1 accident and emergency (major emergency) departments (ED) and all four have type 3 urgent treatment centres. All hospitals have same day emergency care, and two hospitals have rehabilitation units.

Access to the dataset was provided as part of a service evaluation (reference 24HIP09) and included data from patients discharged between 01/2023–02/2024. NHS Health Research Authority ethical approval was not required to conduct this study. In reporting our study, we followed the Strengthening the Reporting of Observational Studies in Epidemiology (STROBE) guidelines (See Appendix 2  for  Supplementary  Table  S1) [20].

### Data preparation

The dataset was prepared by a data analyst at the NHS Trust, including de-identification. Patients who opt out from their data being shared beyond their individual care (e.g. research) as part of the National data opt out were excluded.

Patients were ≥ 65 years (with and without dementia), had a non-elective hospital admission and were admitted to a ward for at least 1 day. Intermediate care (a short term reablement care setting for patients fit for discharge from hospital but requiring additional support/rehabilitation before returning home) was considered as a discharge from the hospital and not included in length of stay or classed as a ward change. Other wards and areas excluded from the total number of ward changes were discharge lounges, virtual wards, theatres and the renal unit.

The following covariates were included in the dataset: age (years), sex, relative deprivation according to the English IMD (quintiles) [19], ethnic group (White [British, Irish, any other white background], Asian [Indian, Pakistani, Bangladeshi, Chinese, any other Asian background], Black [African, Caribbean, any other black background], Mixed, Any other ethnic group), dementia status, number of ward changes, hospital site (Salford, Oldham, Rochdale, Bury), delirium status. The outcomes were length of stay in days (length of stay [LOS]), mortality during admission, discharged to a care home (including the following codes: discharge to assess, local authority or private residential home, NHS nursing home).

Dementia status was derived through International Classification of Diseases, 10^th^ revision (ICD-10) codes from primary and secondary diagnoses. Coding of dementia could include a pre-admission diagnosis and diagnosis during hospitalisation. Where individuals had dementia recorded at one visit but not later visits, dementia status was carried forward. Coding of delirium was also derived through ICD-10 codes, based on screening using the 4 A’s Test (4AT) [21].

**Table 1:** Sample characteristics based on first admission data.

Variable		Dementia diagnosis (n = 2760)	No diagnosis of dementia (n = 16,632)	*p*-value
Age, Mean (SD)		83.4 (7.1)	78.1 (8.0)	<.001
Sex female, N (%)		1584 (57%)	8420 (51%)	<.001
Ethnic Group, N (%)	White Asian Black Mixed Any other ethnic group Missing	2587 (94%) 103 (4%) 14 (<1%) 5 (<1%) 22 (1%) 29 (1%)	15,329 (92%) 815 (5%) 104 (1%) 31 (<1%) 154 (1%) 199 (1%)	.077
Index of Multiple Deprivation, (1 = most deprived, 5 = least deprived)	1 2 3 4 5 Missing	1140 (41%) 619 (22%) 437 (16%) 369 (13%) 188 (7%) 7 (3%)	6220 (37%) 3649 (22%) 2450 (15%) 2759 (17%) 1501 (9%) 53 (3%)	<.001
Dementia subtype^*^	ad Vascular Unspecified Other diseases	1214 (44%) 718 (26%) 767 (28%) 158 (6%)	N/A	N/A
Ward changes, Median (range)		2 (1–9)	2 (1–14)	.745
Readmitted within the timeframe, N (%)		747 (27%)	4212 (25%)	.052
Delirium (ICD code), N (%)		997 (36%)	2397 (14%)	<.001
Length of stay, days^**^ Median (range)		7.69 (1–213)	5.73 (1–418)	<.001
Inpatient mortality, N (%)		295 (11%)	1287 (8%)	<.001
New care home admission, N (%)		294 (11%)	380 (2%)	<.001
Admitted to intermediate care (excluded from number of ward changes)		55 (2%)	356 (2%)	.618

^*^More than one dementia ICD-code could be listed for each individual.

^**^Length of stay excludes individuals that died in hospital.

### Analysis

Descriptive statistics were run to summarise sample characteristics. Group comparisons (those with/without dementia) were made using Pearson’s χ2 (categorical data), independent t test (continuous variables) or Mann–Whitney U test (where data were skewed).

Generalised estimating equations (GEE) were used to run linear and logistic regression models, accounting for some patients having multiple admissions within the time period (standard errors were clustered at patient level). The unadjusted models included three covariates: number of ward changes, a binary indicator indicating that the patient had dementia, and the interaction of ward changes with the dementia indicator. The fully adjusted models included the additional covariates: age, sex, ethnicity (5 groups—reference group white), IMD (5 groups—reference group 1 most deprived) hospital site (4 sites labelled A/B/C/D—reference group site D). In the LOS and discharge destination analyses, people who died during hospitalisation were excluded from analyses. As a sensitivity analysis, delirium (binary indicator) was added to the fully adjusted models. This was separate to the main analyses due to known incompleteness of the delirium data (delirium incidence likely underestimated). For logistic regression analyses, beta coefficients (β) were converted to odds ratios (ORs) by exponentiating the coefficients (i.e. OR = exp(β)). Corresponding 95% confidence intervals were also calculated by exponentiating the lower and upper bounds of the β confidence intervals. We employed listwise deletion, excluding cases with missing values on any of the variables included in the GEE model. A complete-case analysis was therefore performed. Given the LOS data may be skewed, we performed two robustness analyses: (i) we log-transformed the LOS variable and (ii) we excluded the top 5% of LOS values. All analyses were conducted in SPSS version 28.

## Results

The dataset contained 27,140 admissions, 19,392 unique individuals, 2760 of which had dementia recorded. Across the care organisations, Salford contributed 40% of the total sample, Oldham 32%, Bury 24% and Rochdale 5%. Based on data from first admission, patients had a mean age at admission 79.0 (SD 8.1), 52% were female and 92% were White (Table 1 for sample characteristics by dementia status). Appendix 3  Supplementary  Table  S2 summarises variables across all admissions.

The number of wards patients had been admitted to within a single hospital admission ranged 1–14 (1–9 in those with dementia), median number of wards was 2. In the full dataset, there were 4284 admissions from those living with dementia; across these admissions 35% had been on one ward, 50% on two and 15% had been on three or more wards. Across 22,856 admissions from those without dementia, 38% had been on one ward, 44% on two wards and 18% had been on three or more wards.

### Regression analyses

Sample sizes ranged 24,758–27,140 in the unadjusted analyses and 24,425–26,769 in the adjusted analyses.

#### Length of stay

**Figure 1:**
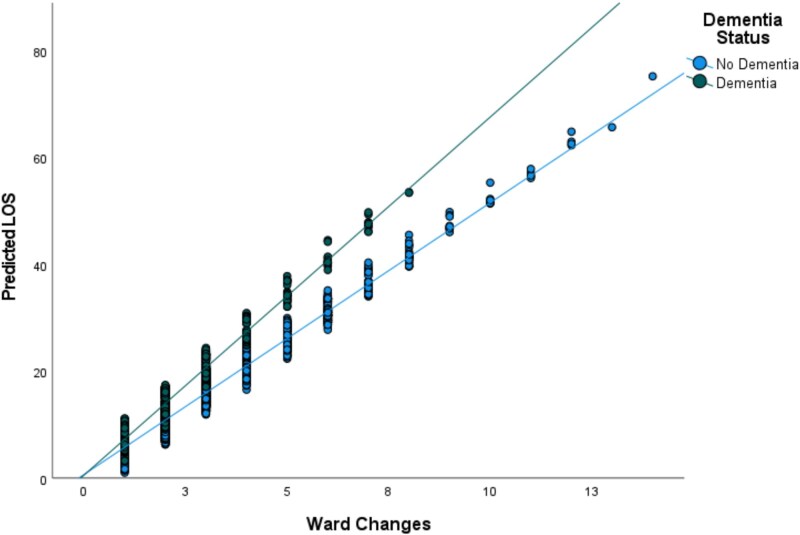
Ward changes and length of stay by dementia status. Footnote: Number of ward changes against predicted length of stay in those with dementia (green) and without dementia (blue).

In unadjusted models, we found that increased ward changes, and the interaction of ward changes and dementia were associated with a longer LOS (Table 2). In fully adjusted models, the results remained the same; ward changes were associated with a longer duration (β = 5.22, 95% CI 4.77–5.67), there was an interaction of ward changes and dementia (β = 1.68, 95% CI 0.86–2.50) but having dementia was not independently associated with LOS (β = −0.44, 95% CI -1.88-1.01). Each ward change increased LOS by 5 days for those without dementia and an additional 1.7 days for those with dementia (Figure 1).

**Table 2:** Regression results.

	Length of stay			Inpatient mortality			Discharged to a care home		
	β (95% Wald CI)	Standard error	*P* value	OR (95% CI)	Standard error	*P* value	OR (95% CI)	Standard error	*P* value
*Unadjusted models*	*N = 24,758*			*N = 27,140*			*N = 24,758*		
Ward changes	**5.03 (4.60, 5.47)**	**0.22**	**<.001**	**1.05 (1.01, 1.09)**	**0.02**	**.020**	**1.23 (1.17, 1.30)**	**0.02**	**<.001**
Dementia	−0.22 (−1.63, 1.19)	0.72	.762	**1.73 (1.32, 2.25)**	**0.13**	**<.001**	**5.21 (4.01, 6.82)**	**0.13**	**<.001**
Interaction of Ward changes and Dementia	**1.66 (0.84, 2.47)**	**0.42**	**<.001**	0.89 (0.78, 1.01)	0.07	.070	0.98 (0.88, 1.09)	0.06	.677
*Fully adjusted models* *^a^*	*N = 24,425*			*N = 26,769*			*N = 24,425*		
Ward changes	**5.23 (4.78, 5.67)**	**0.23**	**<.001**	1.03 (0.99, 1.08)	0.02	.157	**1.27 (1.21, 1.34)**	**0.03**	**<.001**
Dementia	−0.43 (−1.88, 1.02)	0.74	.561	**1.40 (1.07, 1.84)**	**0.14**	**.013**	**4.35 (3.29, 5.75)**	**0.14**	**<.001**
Interaction of Ward changes and Dementia	**1.68 (0.86, 2.50)**	**0.42**	**<.001**	0.90 (0.79, 1.03)	0.07	.136	0.96 (0.85, 1.07)	0.06	.472
*Adjusted for delirium*	*N = 24,425*			*N = 26,769*			*N = 24,425*		
Ward changes	**5.04 (4.60, 5.48)**	**0.23**	**<.001**	1.00 (0.96, 1.05)	0.02	.950	**1.25 (1.19, 1.32)**	**0.03**	**<.001**
Dementia	−0.92 (−2.34, 0.51)	0.73	.208	1.31 (0.99, 1.75)	0.14	.058	**4.14 (3.10, 5.47)**	**0.15**	**<.001**
Interaction of Ward changes and Dementia	**1.41 (0.59, 2.22)**	**0.41**	**<.001**	0.87 (0.76, 1.01)	0.07	.059	0.93 (0.83, 1.05)	0.06	.263

^a^Adjusted for age, sex, ethnicity, deprivation level, hospital site.

**Bold** = significant at *P* < .05.

With regards to the other covariates, older age was associated with a longer LOS, whereas Asian ethnic group, IMD group 5 (least deprived) and being at hospital sites A, B, C were associated with a shorter LOS. See Appendix 4  Supplementary  table  S3 for results for all covariates.

These results were robust to taking the natural logarithm of the LOS and by removing the top 5% of length of stays (Appendix 5  Supplementary  Table  S4). For the natural logarithm results, our model estimates implied that each ward change increases LOS by ~41% for patients without dementia. For those with dementia, the effect of a ward change is more pronounced, increasing LOS by ~55%. Based on mean LOS values from Table 1 (7.69 days for patients with dementia and 5.73 days for those without), these percentage increases translate to approximate LOS increases of 4.2 and 2.3 days, respectively. Likewise, the results from the analysis with ‘extreme’ LOS removed are qualitatively similar, albeit smaller, to the main effects reported here (Appendix 5  Supplementary  Table  S4).

#### Inpatient mortality

In unadjusted models, we found that increased ward changes and dementia increased the likelihood of inpatient mortality, but there was no interaction between the two variables (Table 2). In fully adjusted models, having dementia was still independently associated with an increased likelihood of inpatient mortality (OR 1.40, 95% CI 1.07–1.84) but ward changes were no longer associated (OR 1.03, 95% CI 0.99–1.08), and there was no interaction of ward changes and dementia (OR 0.90, 95% CI 0.79–1.03). However, when hospital site is removed from the fully adjusted model, ward moves are associated with an increased mortality rate.

With regards to the other covariates, older age, being male and being at hospital site C had higher odds of mortality, whereas being at hospital site A and Black ethnic group had lower odds of mortality. See Appendix 4  Supplementary  Table  S3 for results for all covariates.

#### Discharged to a care home

In unadjusted models, we found that increased ward changes and dementia increased the likelihood of being discharged to a care home, but there was no interaction between the two variables (Table 2). In fully adjusted models, these results remained the same: ward changes (OR 1.27, 95% CI 1.21–1.34) and having dementia (OR 4.35, 95% CI 3.29–5.75) were independently associated with being discharged to a care home, but there was no evidence of interaction (OR 0.96, 95% CI 0.85–1.07).

With regards to the other covariates, older age, being at hospital sites A and C had higher odds of being discharged to a care home, whereas Asian ethnic group and IMD groups 5 and 4 (lower levels of deprivation) had lower odds of being discharged to a care home. See Appendix 4  Supplementary  table  S3 for results for all covariates.

#### Sensitivity analyses—models including delirium

Having delirium was associated with poorer outcomes: a longer LOS (β = 5.92, 95% CI 5.34–6.50), increased odds of inpatient mortality (OR 2.03, 95% CI 1.86–2.25) and increased odds of being discharged to a care home (OR 2.05, 95% CI 1.77–2.36). The results concerning ward changes, dementia and their interaction were unchanged, apart from the mortality analysis, where dementia was no longer associated with increased mortality (Table 2).

## Discussion

We found an interaction between the number of ward changes and dementia status on LOS, meaning that as the number of ward changes increase, LOS increases at a greater rate for those people living with dementia. This is in keeping with the known susceptibility of people with dementia to hospital-related harms, such as delirium [22], falls [23] and deconditioning [24, 25]. We did not find an interaction effect between ward changes and dementia in mortality and discharge destination analyses; ward changes increased the likelihood of being discharged to a care home for both people with and without dementia but did not increase the likelihood of inpatient mortality in fully adjusted models.

In interpreting these results, we need to consider patient flow, reasons for ward changes and the possibility of reverse causality, in which people who have a longer LOS may in turn have more ward changes. Across the UK NHS and in the included hospitals in this study, there is a ‘home first’ patient flow model: the ultimate measure of quality for flow is to return people back to their usual place of residence, without harm. Three out of four of the hospitals have an ED and the local policy states that the wait should not exceed 8 from arrival. Transfers to specialist areas from assessment areas, such as the acute medical unit, must be within 4 hours of the need being identified. Ward changes occur for various reasons, including those related to the patient (e.g. changes in clinical condition—escalation or step down), the organisation (e.g. high demand areas), or both (e.g. infection control). Patients with less acute needs may be more likely to outlie on a ward, cared for by a clinical team not based on that ward, prolonging LOS. However, mitigating this, frailty assessment is a part of the local flow policy, to identify the most appropriate inpatient ward for care. It is possible that people who are in hospital longer may have more ward changes; ward changes and LOS likely have a bidirectional relationship whereby ward changes increase LOS, and longer LOS increases the likelihood of further moves.

Our results are similar to research conducted without a dementia focus, in terms of ward changes increasing LOS. One study found that adults moved three or more times had a LOS twice as long (~7 days longer) than those who had been on fewer than three wards [5]. Another study found that atypical transfers (transfers between wards with no overlapping specialities) in hospitalised adults increased LOS by 2.84 days, whereas typical transfers increased their stay by 1.92 days [26]. This estimated increase in LOS is lower than our estimates, however our study focused on older adults, suggesting they are more vulnerable to adverse effects of ward changes. A likely mechanism is the presence of delirium, although in sensitivity analyses, we controlled for the possible confounding effect of delirium, and the interaction effect was still present. However, our data likely underrepresents the true incidence of delirium in the sample, since there is variable compliance in delirium assessment of newly admitted older adults and the timing may be limited to admission only. Research conducted in older adults have found that room changes were strongly associated with new onset delirium [4] and linked to delirium severity [3]. Another reason ward changes increase LOS could be due to changes in the healthcare team, e.g. lack of continuity of care [27].

This study also confirms previous findings that people living with dementia are more likely to be discharged to a care home after hospitalisation, and have an increased likelihood of inpatient mortality [10, 11, 28]. However, when delirium was added to the model, dementia was no longer independently associated with increased mortality, suggesting delirium is driving the association. This aligns with other studies that have demonstrated the adverse effects of delirium in hospital [29, 30]. The relationship between ward changes and mortality and being discharged to a care home have not previously been examined. In the mortality analyses, we found that controlling for hospital site caused the association of ward changes and mortality to disappear. This is likely because the included hospitals differ in the number of ward changes and admission of more acutely unwell patients (e.g. three hospitals have an ED, two hospitals have stroke units). In all fully adjusted models, we controlled for these between-hospital differences.

Strengths of this work include access to a large, real-world dataset. However, routinely collected datasets come with associated limitations, e.g. data quality and completeness. There were known limitations with the available delirium data and coding of dementia may also have been missed, e.g. we amended cases where it was recorded at one visit but not subsequent visits for the same person. The sample lacked diversity in terms of ethnicity, and we did not have the following data: reasons for ward changes, bed moves within the same ward, changes in consultant on the same ward, and lack of information on ward type and being able to adjust for this. We included intensive care and rehabilitation wards in the total number of ward changes, but patients admitted to these wards may differ substantially in their expected LOS. Intermediate care was treated as discharge from hospital and excluded from the number of ward changes. Those admitted to intermediate care would have additional needs that have not been captured by this analysis, since their discharge destination following intermediate care is not included in the analysis, however this is only a small percentage of the sample. A limitation with the interpretation of results concerns reverse causality, as discussed above. These limitations may therefore lead to an imprecise estimation of the true causal effect. However, given the nature of the data that we have, we cannot directly address this here. Future work should address these limitations and confirm these findings across other hospitals. Areas for future work include running a mediation analysis to examine whether delirium mediates the effect of ward changes and dementia on LOS and examining the potential bidirectional relationship between ward changes and LOS.

This work has important implications for clinical practice. Longer hospital stays often result in poorer outcomes such as deconditioning, with people with cognitive impairment being at a heightened risk [24, 25]. In order to reduce LOS and improve patient outcomes, we need a different approach to bed management, particularly for those people living with dementia. Particular attention should be made to ensure continuity of care, minimise bed or ward changes, reduce patients outlying on an alternate speciality ward and to ensure any movements across the hospital are fully justified.

The perspectives of patients in hospital also reinforces that ward changes should be minimised. In qualitative research conducted with older people who were considered a high falls risk, participants reported that bed moves increased stress [6]. Similarly qualitative research conducted with people who spent time on clinically inappropriate hospital wards highlighted that participants disliked being moved between wards numerous times, moves caused distress, disorientation and being moved out of hours caused upset [2]. Failings in communication and safety concerns were also mentioned, such as delays in medication. In another qualitative study, relatives of people with cognitive impairment also commented that movements across hospital caused confusion [31]. Ward changes can also result in lost belongings, which may cause further distress for patients and their families. Missing items such as glasses, hearing aids, dentures can also increase risk of delirium, falls and malnutrition [32].

## Conclusion

Our results, using data from a large UK NHS Trust. suggest that ward changes disproportionately affect LOS, but not mortality or hospital discharge to a care home, in people living with dementia. However, these results come with a caveat of possible reverse causality. Minimising hospital ward transfers may improve outcomes for all older patients but is particularly important in dementia care to minimise the risk of extended hospital stay and the associated harm this can have. Future research should examine the potential bidirectional relationship between ward changes and LOS, and the mediating effect of delirium.

## Supplementary Material

aa-25-660-File002_afaf372
