# Supplementary material for: Do ward changes affect outcomes differently in people living with dementia?
Source: Age Ageing. 2026 Jan 15;55(1):afaf372. doi: 10.1093/ageing/afaf372 (PMC12804177; doi:10.1093/ageing/afaf372)
Supplement: aa-25-660-File002_afaf372 [file aa-25-660-file002_afaf372.docx]

**Supplementary materials**

**Do Ward Changes Affect Outcomes Differently in People Living with Dementia?**

- Appendix 1: Supplementary Methods
- Appendix 2: Table S1. STROBE Statement—checklist of items that should be included in reports of observational studies
- Appendix 3: Table S2. Variables summarized by dementia status across all admissions (27,140)
- Appendix 4: Table S3: Main analyses: results for covariates
- Appendix 5: Table S4: The effect of transforming or censoring the length of stay variable

**Appendix 1: Supplementary Methods**

The Index of Multiple Deprivation (IMD) is the official measure of relative deprivation for small areas in England, produced by the Ministry of Housing, Communities and Local Government. It combines information from seven domains of deprivation—income, employment, education, health, crime, barriers to housing and services, and living environment—to provide an overall score for each neighbourhood (Lower-layer Super Output Area). These domains are weighted and aggregated to identify areas experiencing the highest levels of multiple deprivation across England. These small area measures can then be aggregated to examine different geographical scales. According to the Local Authority District Profiles (file 10 of [1]), out of 317 Local Authority Districts in England, Salford is ranked 20th most deprived, Bury is ranked 110th most deprived, Oldham is ranked 29th most deprived, and Rochdale is ranked 17th most deprived. Therefore, each area is in the most deprived tertile nationally, and three are most deprived decile nationally.

Reference:

1. Ministry of Housing, Communities & Local Government. The English indices of deprivation 2019 (2019).

**Appendix 2: Table S1. STROBE Statement—checklist of items that should be included in reports of observational studies**

|  | | | Item No. | Recommendation | | | | Page  No. | |
| --- | --- | --- | --- | --- | --- | --- | --- | --- | --- |
| **Title and abstract** | | | 1 | (*a*) Indicate the study’s design with a commonly used term in the title or the abstract | | | | 1 | |
|  |  |  |  | (*b*) Provide in the abstract an informative and balanced summary of what was done and what was found | | | | 2 | |
| Introduction | | | | | | | | | |
| Background/rationale | | | 2 | Explain the scientific background and rationale for the investigation being reported | | | | 3 | |
| Objectives | | | 3 | State specific objectives, including any prespecified hypotheses | | | | 3 | |
| Methods | | | | | | | | | |
| Study design | | | 4 | Present key elements of study design early in the paper | | | | 3 | |
| Setting | | | 5 | Describe the setting, locations, and relevant dates, including periods of recruitment, exposure, follow-up, and data collection | | | | 3 | |
| Participants | | | 6 | (*a*) *Cohort study*—Give the eligibility criteria, and the sources and methods of selection of participants. Describe methods of follow-up  *Case-control study*—Give the eligibility criteria, and the sources and methods of case ascertainment and control selection. Give the rationale for the choice of cases and controls  *Cross-sectional study*—Give the eligibility criteria, and the sources and methods of selection of participants | | | | 4 | |
|  |  |  |  | (*b*) *Cohort study*—For matched studies, give matching criteria and number of exposed and unexposed  *Case-control study*—For matched studies, give matching criteria and the number of controls per case | | | | NA | |
| Variables | | | 7 | Clearly define all outcomes, exposures, predictors, potential confounders, and effect modifiers. Give diagnostic criteria, if applicable | | | | 4 | |
| Data sources/ measurement | | | 8* | For each variable of interest, give sources of data and details of methods of assessment (measurement). Describe comparability of assessment methods if there is more than one group | | | | 4 | |
| Bias | | | 9 | Describe any efforts to address potential sources of bias | | | | 5 | |
| Study size | | | 10 | Explain how the study size was arrived at | | | | 4 | |
| Quantitative variables | | 11 | | Explain how quantitative variables were handled in the analyses. If applicable, describe which groupings were chosen and why | | | | 4-5 |  |
| Statistical methods | | 12 | | (*a*) Describe all statistical methods, including those used to control for confounding | | | | 4-5 |  |
|  |  |  |  | (*b*) Describe any methods used to examine subgroups and interactions | | | | 5 |  |
|  |  |  |  | (*c*) Explain how missing data were addressed | | | | 5 |  |
|  |  |  |  | (*d*) *Cohort study*—If applicable, explain how loss to follow-up was addressed  *Case-control study*—If applicable, explain how matching of cases and controls was addressed  *Cross-sectional study*—If applicable, describe analytical methods taking account of sampling strategy | | | | NA |  |
|  |  |  |  | (*e*) Describe any sensitivity analyses | | | | 4 |  |
| Participants | | 13* | | (a) Report numbers of individuals at each stage of study—eg numbers potentially eligible, examined for eligibility, confirmed eligible, included in the study, completing follow-up, and analysed | | | | 5 |  |
|  |  |  |  | (b) Give reasons for non-participation at each stage | | | | NA |  |
|  |  |  |  | (c) Consider use of a flow diagram | | | | NA |  |
| Descriptive data | | 14* | | (a) Give characteristics of study participants (eg demographic, clinical, social) and information on exposures and potential confounders | | | | 5 and Table 1 |  |
|  |  |  |  | (b) Indicate number of participants with missing data for each variable of interest | | | | Table 1 |  |
|  |  |  |  | (c) *Cohort study*—Summarise follow-up time (eg, average and total amount) | | | | NA |  |
| Outcome data | | 15* | | *Cohort study*—Report numbers of outcome events or summary measures over time | | | | Table 1 |  |
|  |  |  |  | *Case-control study—*Report numbers in each exposure category, or summary measures of exposure | | | | NA |  |
|  |  |  |  | *Cross-sectional study—*Report numbers of outcome events or summary measures | | | | NA |  |
| Main results | | 16 | | (*a*) Give unadjusted estimates and, if applicable, confounder-adjusted estimates and their precision (eg, 95% confidence interval). Make clear which confounders were adjusted for and why they were included | | | | 5-6 and Table 2 |  |
|  |  |  |  | (*b*) Report category boundaries when continuous variables were categorized | | | | NA |  |
|  |  |  |  | (*c*) If relevant, consider translating estimates of relative risk into absolute risk for a meaningful time period | | | | NA |  |
| Other analyses | 17 | | Report other analyses done—eg analyses of subgroups and interactions, and sensitivity analyses | | |  | | 6 |  |
| Discussion | | | | | | | | |  |
| Key results | 18 | | Summarise key results with reference to study objectives | | |  | | 7 |  |
| Limitations | 19 | | Discuss limitations of the study, taking into account sources of potential bias or imprecision. Discuss both direction and magnitude of any potential bias | |  | | | 7 |  |
| Interpretation | 20 | | Give a cautious overall interpretation of results considering objectives, limitations, multiplicity of analyses, results from similar studies, and other relevant evidence | |  | | 7-8 | |  |
| Generalisability | 21 | | Discuss the generalisability (external validity) of the study results | |  | | 8 | |  |
| Other information | | |  | | | | | |  |
| Funding | 22 | | Give the source of funding and the role of the funders for the present study and, if applicable, for the original study on which the present article is based | |  | | Funding section of manuscript | |  |

*Give information separately for cases and controls in case-control studies and, if applicable, for exposed and unexposed groups in cohort and cross-sectional studies.

**Note:** An Explanation and Elaboration article discusses each checklist item and gives methodological background and published examples of transparent reporting. The STROBE checklist is best used in conjunction with this article (freely available on the Web sites of PLoS Medicine at http://www.plosmedicine.org/, Annals of Internal Medicine at http://www.annals.org/, and Epidemiology at http://www.epidem.com/). Information on the STROBE Initiative is available at www.strobe-statement.org.

**Appendix 3: Table S2. Variables summarized by dementia status across all admissions (27,140)**

| Variable |  | Dementia diagnosis (n=4284) | No diagnosis of dementia (n=22,856) |
| --- | --- | --- | --- |
| Ward changes, Median (range) |  | 2 (1-9) | 2 (1-14) |
| Delirium (ICD code), N (%) |  | 1595 (37%) | 3547 (16%) |
| Length of stay, days  Median (range) |  | 7.66 (1-213) | 6.00 (1-418) |
| Inpatient mortality, N (%) |  | 476 (11%) | 1906 (8%) |
| New care home admission, N (%) |  | 463 (11%) | 579 (3%) |

**Appendix 4: Table S3. Main analyses: results for covariates**

|  | **Length of stay** | | | **Inpatient mortality** | | | **Discharged to a care home** | | |
| --- | --- | --- | --- | --- | --- | --- | --- | --- | --- |
|  | **β (95% Wald CI)** | **Standard error** | **P value** | **OR (95% CI)** | **Standard error** | **P value** | **OR (95% CI)** | **Standard error** | **P value** |
| *Main analyses* | *N=24,425* | | | *N=26,769* | | | *N=24,425* | | |
| Age | **0.06 (0.04-0.08)** | **0.01** | **<.001** | **1.04 (1.03, 1.04)** | **0.00** | **<.001** | **1.05 (1.04, 1.06)** | **0.00** | **<.001** |
| Sex (Male) | 0.27 (-0.09-0.64 | 0.19 | .142 | **1.21 (1.11, 1.31)** | **0.04** | **<.001** | 0.96 (0.84, 1.11) | 0.07 | .552 |
| Ethnicity  Asian  Black  Mixed  Any other  White (reference group) | **-1.40 (-2.13, -0.67)**  0.46 (-1.50, 2.42)  -1.08 (-3.48, 1.33)  1.38 (-1.40, 4.17) | **0.37**  1.00  1.23  1.42 | **<.001**  .643  .381  .330 | 0.87 (0.69, 1.08)  **0.42 (0.19, 0.93)**  1.01 (0.36, 2.83)  1.09 (0.69, 1.75) | 0.12  **0.41**  0.53  0.24 | .217  **.034**  .986  .696 | **0.18 (0.10, 0.35)**  0.15 (0.02, 1.16)  1.49 (0.39, 5.81)  0.53 (0.20, 1.39) | **0.33**  1.04  0.69  0.49 | **<.001**  .070  .561  .199 |
| IMD  5 (least deprived)  4  3  2  1 (most deprived – reference group) | **-0.75 (-1.43, -0.07)**  -0.09 (-0.65, 0.47)  0.03 (-0.56, 0.61)  0.39 (-0.10, 0.87) | **0.35**  0.28  0.30  0.25 | **.032**  .748  .930  .116 | 0.90 (0.77, 1.07)  0.91 (0.80, 1.04)  1.01 (0.89, 1.15)  0.92 (0.83, 1.04) | 0.08  0.07  0.07  0.06 | .262  .167  .917  .190 | **0.58 (0.42, 0.79)**  **0.80 (0.64, 0.99)**  1.14 (0.93, 1.39)  1.13 (0.95, 1.34) | **0.16**  **0.11**  0.10  0.09 | **<.001**  **.045**  .210  .159 |
| Hospital site  A  B  C  D (reference group) | **-3.80 (-4.29, -3.31)**  **-2.58 (-3.03, -2.12)**  **-3.60 (-4.13, -3.06)** | **0.25**  **0.23**  **0.27** | **<.001**  **<.001**  **<.001** | **0.41 (0.31, 0.55)**  0.96 (0.87, 1.06)  **1.17 (1.05, 1.31)** | **0.15**  0.05  **0.05** | **<.001**  .456  **.003** | **2.59 (2.05, 3.29)**  0.95 (0.79, 1.13)  **1.27 (1.07, 1.51)** | **0.12**  0.09  **0.09** | **<.001**  .539  **.006** |

**Bold** = significant at p<.05

**Appendix 5: Table S4. The effect of transforming or censoring the length of stay variable**

|  | **Panel A: Length of stay (as in paper)** | | | **Panel B: Length of stay (as log(LOS))** | | | **Panel C: Length of stay (as in paper but with outliers removed)** | | |  |
| --- | --- | --- | --- | --- | --- | --- | --- | --- | --- | --- |
|  | **β (95% Wald CI)** | **Standard error** | **P value** | **β (95% Wald CI)** | **Standard error** | **P value** | **β (95% Wald CI)** | **Standard error** | **P value** |  |
| *Unadjusted models* | *N=24,758* | | | *N=24,758* | | | *N=23,617* | | |  |
| Ward changes | **5.03 (4.60, 5.47)** | **0.22** | **<.001** | **0.40 (0.38, 0.41)** | **0.01** | **<.001** | **2.73 (2.60, 2.86)** | **0.07** | **<.001** |  |
|  |  |  |  |  |  |  |  |  |  |  |
| Dementia | -0.22 (-1.63, 1.19) | 0.72 | .762 | -0.02 (-0.10, 0.06) | 0.04 | .694 | -0.55 (-1.23, 0.12) | 0.34 | .106 |  |
| Interaction of Ward changes and Dementia | **1.66 (0.84, 2.47)** | **0.42** | **<.001** | **0.13 (0.09, 0.17)** | **0.02** | **<.001** | **1.24 (0.86, 1.61)** | **0.19** | **<.001** |  |
| *Fully adjusted models** | *N=24,425* | | | *N=24,425* | | | *N=23,298* | | |  |
| Ward changes | **5.23 (4.78, 5.67)** | **0.23** | **<.001** | **0.41 (0.40, 0.43)** | **0.01** | **<.001** | **2.84 (2.71, 2.97)** | **0.07** | **<.001** |  |
| Dementia | -0.43 (-1.88, 1.02) | 0.74 | .561 | -0.06 (-0.14, 0.02) | 0.04 | .131 | **-0.96 (-1.65, -0.28)** | **0.35** | **.006** |  |
| Interaction of Ward changes and Dementia | **1.68 (0.86, 2.50)** | **0.42** | **<.001** | **0.14 (0.10, 0.0.18)** | **0.02** | **<.001** | **1.27 (0.89, 1.64)** | **0.19** | **<.001** |  |
| *Adjusted for delirium* | *N=24,425* | | | *N=24,425* | | | *N=23,298* | | |  |
| Ward changes | **5.04 (4.60, 5.48)** | **0.23** | **<.001** | **0.40 (0.38, 0.41)** | **0.01** | **<.001** | **2.75 (2.63, 2.88)** | **0.06** | **<.001** |  |
|  |  |  |  |  |  |  |  |  |  |  |
| Dementia | -0.92 (-2.34, 0.51) | 0.73 | .208 | **-0.10 (-0.18, -0.02)** | **0.04** | **.012** | **-1.21 (-1.89, -0.52)** | **0.35** | **<.001** |  |
| Interaction of Ward changes and Dementia | **1.41 (0.59, 2.22)** | **0.41** | **<.001** | **0.11 (0.08, 0.15)** | **0.02** | **<.001** | **1.08 (0.71, 1.45)** | **0.19** | **<.001** |  |

**Bold** = significant at p<.05

*Adjusted for age, sex, ethnicity, deprivation level, hospital site
